# A Data Correction Algorithm for Low-Frequency Floating Car Data

**DOI:** 10.3390/s18113639

**Published:** 2018-10-26

**Authors:** Bijun Li, Yuan Guo, Jian Zhou, Yi Cai

**Affiliations:** 1 State Key Laboratory of Information Engineering in Surveying, Mapping, and Remote Sensing, Wuhan University, Wuhan 430079, China; Lee@whu.edu.cn (B.L.); guoyuan@whu.edu.cn (Y.G.); clarify@whu.edu.cn (Y.C.); 2 Engineering Research Center for Spatio-Temporal Data Smart Acquisition and Application, Ministry of Education of China, Wuhan University, Wuhan 430079, China

**Keywords:** data correction, map matching, OpenStreetMap, floating car

## Abstract

The data collected by floating cars is an important source for lane-level map production. Compared with other data sources, this method is a low-cost but challenging way to generate high-accuracy maps. In this paper, we propose a data correction algorithm for low-frequency floating car data. First, we preprocess the trajectory data by an adaptive density optimizing method to remove the noise points with large mistakes. Then, we match the trajectory data with OpenStreetMap (OSM) using an efficient hierarchical map matching algorithm. Lastly, we correct the floating car data by an OSM-based physical attraction model. Experiments are conducted exploiting the data collected by thousands of taxies over one week in Wuhan City, China. The results show that the accuracy of the data is improved and the proposed algorithm is demonstrated to be practical and effective.

## 1. Introduction

With the development of self-driving vehicles, lane-level maps have drawn much attention from researchers, internet firms, and carmakers. Currently, lane-level map generation methods mainly include the following three approaches: an integrated navigation system (INS) with three-dimensional (3D) Lidar [1,2]; a vision-based approach [3]; and a floating car-based approach [4,5,6]. The precision of the map can reach the centimeter level using the Lidar approach, but the cost of the devices and production is the most expensive of the three approaches. The vision-based approach captures the map information by monocular camera or stereo camera, however, the quality of the map is influenced by the condition of the light to a great degree; moreover, the cost of production is also high. The cost of the map is low if the data is collected by a floating car. However, the positional accuracy of global positioning system (GPS) traces can only reach 5–30 m because GPS traces are prone to errors due to the multipath effect and the loss of satellite signals. Therefore, it is a challenging task to produce a map with floating car data.

In this paper, we propose a new data correction method for low-frequency floating vehicle data. We developed an adaptive density optimization method to remove a fraction of the noise points by using a Delaunay triangulation network to construct clusters of points. As OpenStreetMap (OSM) has become one of the most successful projects in Volunteered Geographic Information (VGI) project, it is free and has a range of applications. We attempt to match GPS traces with OSM maps and correct the GPS traces by an OSM-based physical attraction model.

In summary, the main contributions of this paper include the following: (1) we improved the spatial–temporal (ST)-matching algorithm through a hierarchical method to match the floating car data and OSM map, by which the accuracy and efficiency of the algorithm have been improved; (2) we used a physical attraction model to correct the GPS points; (3) we proved that it is feasible to improve the accuracy of the GPS points using the OSM map; and (4) we showed that the improvement in accuracy of the GPS traces was helpful for the production of lane-level maps.

The remainder of the paper is structured as follows: In Section 2, we describe the related work of data correction; in Section 3 we discuss the data correction algorithm; in Section 4 we present the experimental result of the algorithm; and finally we present our conclusions in Section 5.

## 2. Related Work

Methods for correcting floating car data include the following: (1) clusters; (2) filters; and (3) projecting the GPS points onto an existing map by map matching.

Approaches for removing noise points with clusters have been used by many researchers. Among them, kernel density methods have often been used to build the probability function of the GPS points, and the points with a low spatial density are thus eliminated as outliers [7,8]. Biagioni and Eriksson [9] proposed a single sample point density estimate using a kernel density estimator with a Gaussian kernel and extracted an initial road network based on the density estimate. In References [10,11,12], the authors clustered the GPS data into regions based on the similarity of the position and direction and filtered out the noise by using the average of the clusters. However, the approaches mentioned above can only remove a small number of the noise points; the precision cannot be completely improved.

Various filtering techniques can also be used to smooth the noise of GPS trajectories. In Reference [13], mean and median filters were applied to smooth the noise. These two filters are similar, in that the median filter merely replaces the mean in the mean filter with the median. The mean and median filters are simple but sensitive to outliers. More complex filters have also been used to correct GPS data, such as a Kalman filter or a particle filter [13,14,15,16]; however, they are mainly used in high-frequency GPS data processing.

Map matching algorithms for low-frequency data include both local and global algorithms [17]. Local matching algorithms usually match GPS points based on the distance, heading, speed, topology, and shortest path [18,19]. However, the accuracy of local algorithms is generally low and sensitive to the sampling frequency. Global algorithms match the entire trajectory with the road, based on geometric similarity [20,21]. The limitations of global algorithms are highly time consuming and computationally costly. Zhang et al. [22] matched traces with an existing road map based on three features: the distance, direction, and angle between the trace and map. They estimated a new centerline by modeling the traces with a Gaussian distribution, but the precision of their algorithm can only reach to road level.

Different from the methods mentioned above, this paper corrects GPS points in two steps: we first remove part of the noise by using an adaptive density optimization method, and then correct the points through a physical attraction model and a matched OSM map.

## 3. Data Correction Algorithm

**Figure 1:**
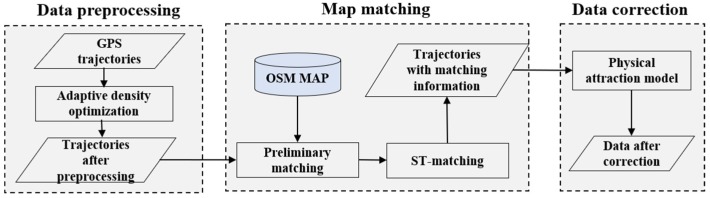
Overview of the proposed algorithm.

The processes of our proposed algorithm include the following three steps, as shown in Figure 1: data preprocessing, map matching, and data correction. The following subsections detail each of these steps.

### 3.1. Problem Statement

**Figure 2:**
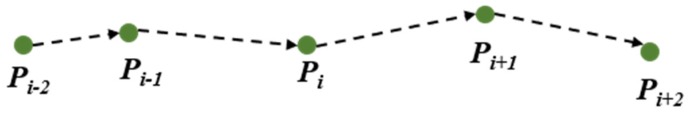
A trajectory of the GPS points. The green dots represent the GPS points and arrows represent the driving direction of floating car. $P_{i}$ is the ID of the GPS point.

In the floating car system, the location of a car is recorded by the GPS. A trajectory is a collection of GPS points arranged in a time sequence, $T=\left\{P_{1},P_{2},\ldots \ldots P_{n}\right\}$, as shown in Figure 2. Each of the points $P_{i}$ has attributes $P_{i}=\left\{x_{i},y_{i},t_{i}\right\}$, where ($x_{i}$, $y_{i}$) are the longitude and latitude of the point, respectively, and *t_i_* is the time the point was collected.

The positional accuracy of GPS traces can reach 5–30 m, but the error will increase when the satellite signals are obstructed by tall buildings, trees, and tunnel. Therefore, it is necessary to remove the points with large error first. However, it is still hard to generate a lane-level map using this GPS data because some points appear on the opposite road, which will influence the results of the lane position. The aim of this paper is to correct the GPS points to the right location by a physical attraction model based on the OSM map information.

### 3.2. Trajectory Preprocessing

**Figure 3:**
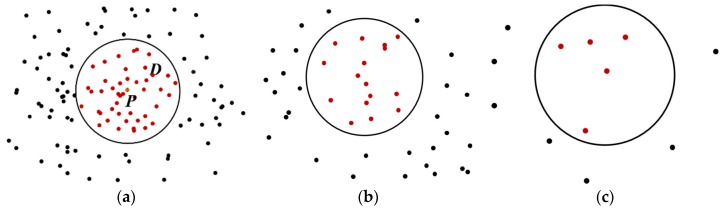
Areas with different point densities. *D* represents the buffer of central point *P*. The circle is the range of buffer *D*. Red dots represent the GPS points inside *D*, and black dots are the GPS points outside *D*. (**a**) The points in the center of a city have more neighboring points; (**b**) the points out of the center of a city with lower density; and (**c**) the points at the edge of a city, which might be recognized as noise.

To remove the noise points with a large error, the trajectory should be preprocessed first. In general, points with fewer neighboring points are identified as outliers. However, as shown in Figure 3, there is a big difference in density between different roads because of the different grades of the roads and the divisions of urban zoning. The density of the points in a city center is larger than that of the points at the edge of a city. If we were to use the same threshold to distinguish the noise in both the high and low density areas, the correct points would be recognized as noise in the areas with low density. Therefore, it is necessary to choose an adaptive density threshold to preprocess the data.

The density of a GPS point can be described by the null distribution [23]. The null distribution of point *P* can be defined as follows:

(1) $$P\left(N\left(S\right)=k\right)=\frac{\lambda^{k}{\left|B\right|}^{k}}{k!}\cdot e^{-\lambda \left|B\right|}$$

(2) $$P(X<n_{i})=\sum_{j=0}^{n_{i}-1}\frac{e^{-\hat{\lambda }}{\hat{\lambda }}^{j}}{j!}$$

(3) $$\hat{\lambda }=\frac{N_{D}}{\left|D\right|}$$

(4) $$P\left(X\geq n_{i}\right)=1-P(X<n_{i})$$

Equation (1) is the expression of the null distribution, where *N*(*S*) is the number of points for a spatial point dataset SD, *λ* is the intensity of the point dataset, and |*B*| is the area of SD. In this paper, we calculate the density of GPS point *P* through the number of points in buffer *D*. As shown in Figure 2, the probability of the number of points *X* < *n_i_* in a buffer *D* can be calculated via Equation (2). The value of $\hat{\lambda }$ can be calculated by Equation (3), where *N_D_* is the number of points in buffer *D*, and |*D*| is the area of buffer *D*. Through Equation (4), we can calculate the probability of *X* ≥ *n_i_* in buffer *D*.

**Figure 4:**
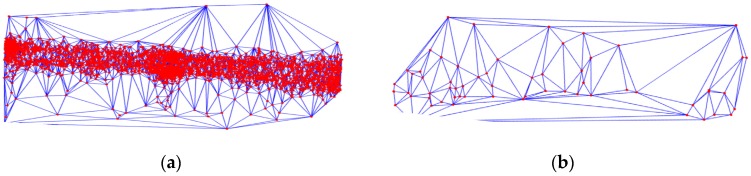
The Delaunay triangulation network of GPS points. The red dots represent the GPS points and the blue lines are the edges of the Delaunay triangulation network. (**a**) in the center of the city, the length and variance of edges in the Delaunay triangulation network are small, so the value of *R_S_* is small; (**b**) at the edge of the city, the density of points is small, so the length and variance of the edges in the Delaunay triangulation network is large. As a result, the value of *R_S_* is large.

As already noted, on a different road, the density of GPS points is different. Therefore, we used a Delaunay triangulation network to calculate the radius of the buffer:

(5) $$R_{S}=Mean\left(AT\right)+Variation\left(AT\right)$$

where *Mean*(*AT*) is the average value of the length of the sides in the Delaunay triangulation network and *Variation*(*AT*) is the variance of the lengths of the sides. In the center of the city, the density of points is large, so the value of *R_S_* is small. In contrast, the value of *R_S_* is large at the edge of the city, as shown in Figure 4.

### 3.3. Hierarchical Map Matching Algorithm (HST-Matching)

After the preprocessing, the points with large position error have been removed. However, the accuracy of GPS trajectory still cannot meet the requirement of the lane-level map. In this section, we propose hierarchical map matching (HST-Matching) method, by improving the ST-matching algorithm [17], to match low-frequency floating car data with the OSM map. The HST-Matching algorithm consists of two parts: preliminary matching and ST-matching.

The OSM map, as crowdsourced geographic data, is one of the current research trends. The main advantage is its low cost. From the literature [24,25,26], we know that most of the OSM map data have high accuracy. However, the precision of the GPS points in the floating car measurement is 5–30 m. Therefore, we can attempt to match the GPS points with the OSM map to improve the accuracy of the GPS traces.

The OSM map consists of three parts: nodes, ways, and relations [27]. The nodes can be used to define standalone point features or the shape of a way. The ways are used to represent the linear features, for example, rivers and roads. A way consists of an ordered list of nodes between 2 and 2000 and is defined as a polyline. A relation is used to describe the relationship between two or more data elements, including turn restrictions. The ways in the OSM map not only include the roads, but also rivers, subways, and the boundary river. We only selected the types of roads on which vehicles can drive, including motorway, trunk, primary, secondary, tertiary, service, and residential roads.

**Figure 5:**
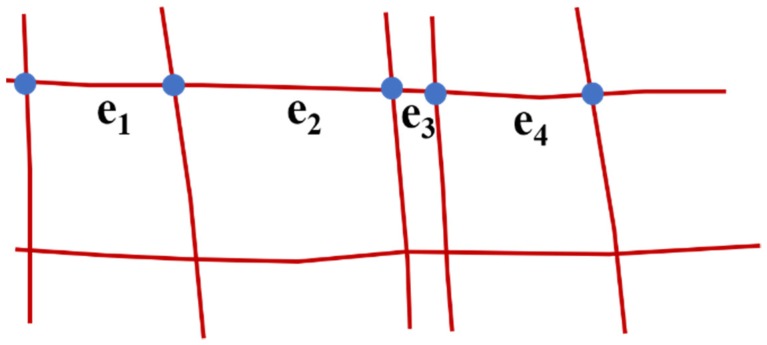
The segments in the OSM map. The red lines represent the roads of OSM, the blue points are the start or end points of segments, and *e_i_* means the segment of road.

A complete road is divided into many segments in the OSM map, $R=\left\{e_{1},e_{2},\ldots \ldots e_{n}\right\}$. Each segment contains a starting point, $e_{i}.start$, an endpoint, $e_{i}.end$, and the nodes that control the shape of the road, $e_{i}.control$, as shown in Figure 5.

Before we introduce the map matching algorithm, it is necessary to describe the assumptions used in this paper.

**Assumption** **1.** *The vehicle runs on the roads, so the trajectory can be matched to at least one road.*

**Assumption** **2.** *The path of the car tends to be direct, rather than a roundabout route. This means that the matching path between two GPS points will most likely be the shortest path.*

#### 3.3.1. Preliminary Matching

In the preliminary matching step, we generated the buffer of each point *P_i_*, 1 ≤ *i* ≤ *n*, with radius *r* in a trajectory *T* = {*P*_1_, *P*_2_ … *P_n_*} to retrieve the candidate segments and candidate points of *P_i_*. As we had already preprocessed the trajectory, the noises with the largest deviations had been removed. The radius of the buffer was set as 30 m.

**Figure 6:**
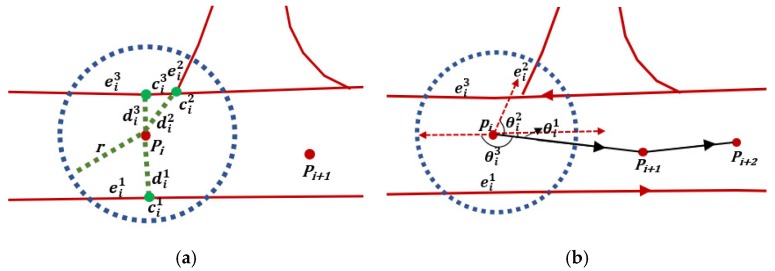
(**a**) *P_i_* means the ID of GPS point and $e_{i}^{j}$ represents the candidate segment of road. $d_{i}^{j}$ means the distance between *P_i_* and the candidate segment. $c_{i}^{j}$ is ID of the candidate point of *P_i_*. The red lines represent the roads of OSM, and blue dashed lines represent the buffer of center point *P_i_*. Green dashed lines represent the distance between *P_i_* and candidate points. The red dots mean the GPS points, and the green dots are the candidate points of *P_i_*. In the buffer of point *P_i_*, there are three segments with which it intersects. (**b**) The red dashed lines indicate the direction of the candidate segments; $\theta_{i}^{j}$ means the angle differences; only segment $e_{i}^{1}$ remains after being filtered out by a threshold.

An example is shown in Figure 6a. In the buffer of point *P_i_*, there are three candidate segments $P_{i}=\left\{e_{i}^{1},e_{i}^{2},e_{i}^{3}\right\}$. The distances between *P_i_* and the candidate segments are $\left\{d_{i}^{1},d_{i}^{2},d_{i}^{3}\right\}$, and the candidate points of *P_i_* are $\left\{c_{i}^{1},c_{i}^{2},c_{i}^{3}\right\}$. As azimuth information in floating car data is lacking, we calculate the angle differences between the vector connecting points *P_i_* and *P_i_*_+1_ and the candidate segments direction $\left\{e_{i}^{1},e_{i}^{2},e_{i}^{3}\right\}$. As shown in Figure 6b, the angle differences are $\left\{\theta_{i}^{1},\theta_{i}^{2},\theta_{i}^{3}\right\}$. We use a threshold $T_{\theta }$ to filter out parts of candidate segments. If $\theta_{i}^{j}>T_{\theta }$, the corresponding segment of angle $\theta_{i}^{j}$ is removed. If only one candidate segment, $e_{i}^{1}$, remains, point *P_i_* is counted as a high-confidence tracking point (HCTP) according to Assumption 1 and segment $e_{i}^{1}$ is the matched road of point *P_i_*. Algorithm 1 shows the details of the preliminary matching procedure.

**Algorithm 1** Preliminary Matching Algorithm
**Input:**
Trajectory *P*_1_ → *P*_2_ … → *P_n_*; OSM road network *R*
**Output:**
HCTPlist; Candidate matched points list $c_{i}^{1}$, $c_{i}^{2}$ … $c_{i}^{j}$1:Initialize HCTPlist and CanditateList as empty list;2:**for***i* = 1 to *n*
**do**3:  C = GetCandidate (*P_i_*, *R*, *r*); //get the candidates within radius *r*4:  **for**
*j* = 1 to C.count **do**5:  $\theta_{i}^{j}$ = |azi_*P_i_*-azi_$c_{i}^{j}$|;6:  **if**
$\theta_{i}^{j}$ < $T_{\theta }$
**then**7:   CandidatedList.add ($c_{i}^{j}$);8:  **end if**9:  **end for**10:  **if** CandidateList.count == 1 **then**11:  HCTPlist.add (*P_i_*);12:  **end if**13:
**end for**


If a point was counted as high-confidence tracking point (HCTP), we only retained the candidate segment which meets the requirement of threshold $T_{\theta }$; other candidate segments will be deleted. There is no need to calculate further. This can reduce the uncertainty and running time of the matching algorithm. However, for other points which do not count as HCTPs, we calculated it by the ST-matching algorithm.

#### 3.3.2. Spatial–Temporal Matching

After the preliminary matching step, some points will have matched with the OSM map. However, there will be many points left to be matched. We choose an ST-matching algorithm to match these points [17,28]. ST-matching is a stable global optimization matching algorithm which can integrate the geometrical, topological, and speed information of traces and map. It includes two steps: spatial analysis and temporal analysis. First, the observation probability is calculated by identifying the shortest distance between the GPS points and the candidate points. Then, the transmission probability is estimated by comparing the shortest path of the GPS points and the candidate points. The temporal probability is calculated by the cosine distance to measure the similarity between the actual speed of the path and the road speed constraints. Finally, the candidate segment with the largest value is considered to be the final matching result.

We improve this algorithm in three ways. In addition to the HCTPs, we give different weights to the observation and transmission probabilities. Considering that the shortest path is more reliable than the position accuracy of GPS. Therefore, the transmission probability has a higher reliability than the observation probability. Its weight should be set to be larger than the observation probability. Beyond this, the road speed constraint should be a range rather than a simple value. Therefore, we use a new method to calculate the temporal value. This is calculated by comparing the probability of the actual speed and the road speed constraint range.

##### Spatial Analysis

According to Reference [9], the position error of the GPS can be described with a normal distribution, and the formula of the observation probability can be calculated via:

(6) $$O\left(c_{i}^{j}\right)=\frac{1}{\sqrt{2\pi }\sigma }e^{-\frac{{\left(d_{i}^{j}-\mu \right)}^{2}}{2\sigma^{2}}}$$

where $d_{i}^{j}$ is the distance between point *P_i_* and candidate segment $e_{i}^{j}$, $d_{i}^{j}=dist\left(c_{i}^{j},P_{i}\right)$, and μ and σ are the mean and variance value of normal distribution.

**Figure 7:**
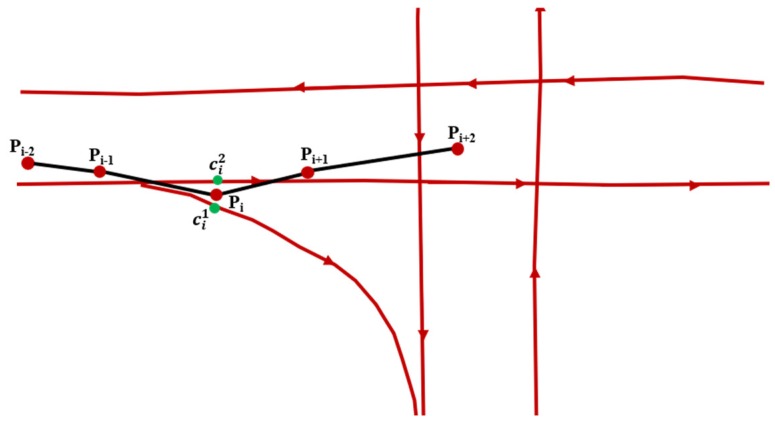
An example of the transmission probability. *P_i_* means the ID of GPS point and $c_{i}^{j}$ represent the ID of candidate point. Red dots mean the GPS points of the trajectory and the black lines are the trajectory of floating car. The red lines represent the OSM roads and the green dots indicate the candidate points of *P_i_*.

Because of the position error of the GPS, it is not enough to only consider the Euclidean distance between the GPS trace and the segment. For example, as shown in Figure 7, there are two candidate points for *P_i_*, $\left(c_{i}^{1},c_{i}^{2}\right)$. The observation probabilities of these two candidate points are equal. Obviously, the correctly matched point should be $c_{i}^{2}$ according to Assumption 2. Hence, topological information is important for map matching, by which we can exclude certain points. The formula of the transmission probability is shown in Equation (7):

(7) $$T\left(c_{i-1}^{k}\to c_{i}^{j}\right)=\frac{dis\left(P_{i-1},P_{i}\right)}{S_{\left(c_{i-1}^{k}\to c_{i}^{j}\right)}}$$

where $dis\left(P_{i-1},P_{i}\right)$ is the Euclidean distance between tracking points $P_{i-1}$ and $P_{i}$ and $S_{\left(c_{i-1}^{k}\to c_{i}^{j}\right)}$ represents the shortest path between candidate points $c_{i-1}^{k}$ and $c_{i}^{j}$. There are many algorithms for the shortest path computation, such as the Dijkstra, Floyd, and A* algorithms [29]. Considering the efficiency of the algorithms, we choose the A* algorithm to calculate the shortest path.

Combining Equations (6) and (7), the spatial analysis function is calculated as follows:

(8) $$F_{s}\left(c_{i-1}^{k}\to c_{i}^{j}\right)=w_{1}O\left(c_{i}^{j}\right)+w_{2}T\left(c_{i-1}^{k}\to c_{i}^{j}\right),\;2\leq i\leq n$$

where $w_{1}$ and $w_{2}$ represent the weight of the observation and the transmission probabilities, respectively, and $w_{1}+w_{2}=1$.

##### Temporal Analysis

The spatial analysis can match the GPS trace to the OSM map in most cases. However, there are lots of elevated roads in China, and the shape and location of the elevated roads are similar to the roads underneath them. Therefore, it is difficult to match the GPS trace using only spatial analysis. However, as shown in Table 1, the road speed constraints are different for the elevated roads than for other roads. Therefore, it is feasible to use the road speed constraint information to refine our analysis. We calculate the probability of the actual speed of a vehicle in different road speed constraint ranges, as shown in the equations below:

(9) $$F_{t}\left(c_{i-1}^{k}\to c_{i}^{j}\right)=\int_{\bar{V}.min}^{\bar{V}.max}\frac{1}{\sqrt{2\pi }R.\sigma }e^{-\frac{{\left(\bar{V}\left(c_{i-1}^{k}\to c_{i}^{j}\right)-R.u\right)}^{2}}{2R.\sigma^{2}}}$$

(10) $$\bar{V}\left(c_{i-1}^{k}\to c_{i}^{j}\right)=\left[\bar{V}.min,\bar{V}.max\right]$$

(11) $$\bar{V}.min=\frac{S_{\left(c_{i-1}^{k}\to c_{i}^{j}\right)}}{\Delta t_{\left(i-1\to i\right)}}-\tau$$

(12) $$\bar{V}.max=\frac{S_{\left(c_{i-1}^{k}\to c_{i}^{j}\right)}}{\Delta t_{\left(i-1\to i\right)}}+\tau$$

where R.u and R.σ represent the mean value and variance of the candidate road speed, respectively, and $\bar{V}\left(c_{i-1}^{k}\to c_{i}^{j}\right)$ is the average speed of the car from point $c_{i-1}^{k}$ to $c_{i}^{j}$, $\Delta t_{\left(i-1\to i\right)}$ is the time interval from point $c_{i-1}^{k}$ to $c_{i}^{j}$, and τ is the calculation error of the speed.

Combining Equations (8) and (9), the final ST-matching function is:

(13) $$F\left(c_{i-1}^{k}\to c_{i}^{j}\right)=F_{s}\left(c_{i-1}^{k}\to c_{i}^{j}\right)\times F_{t}\left(c_{i-1}^{k}\to c_{i}^{j}\right)2\leq i\leq n.$$

**Figure 8:**
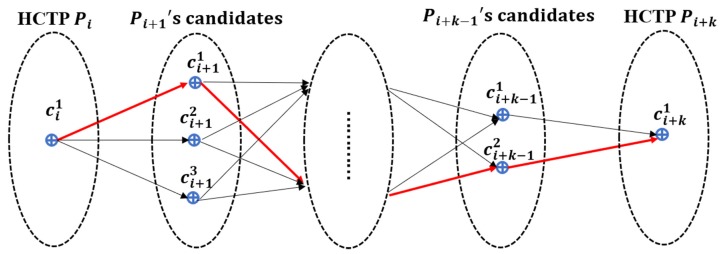
The matching result of the ST-algorithm. *P_i_* means the ID of GPS point and $c_{i}^{j}$ is the ID of candidate point. The points *P_i_* and *P_i+k_* are the HCTP points, so there is only one candidate point. The black arrows represent the candidate matching path from *P_i_* to *P_i_*_+1_, and the red arrows represent the final matching results.

After the HST-matching steps, the value of $F\left(c_{i-1}^{k}\to c_{i}^{j}\right)$ should be calculated for each candidate point. We select the highest score as the matching result between two HCTPs, as shown by the red line in Figure 8. Algorithm 2 shows the process of ST-matching.

**Algorithm 2** Spatial and Temporal Matching Algorithm
**Input:**
HCTPlist *P_i_*, *P_i+k_*; CandidateList $c_{i}^{1}$, $c_{i}^{2}$ … $c_{i}^{j}$; Trajetory *P_i+_*_1_ → *P_i+_*_2_ … → *P_i+k_*
**Output:**
OSM-WayID-List;1:Initialize OSM-WayID-List as empty list;2:**for** each $c_{i}^{1}$ and $c_{i+k}^{1}$
**do**3:  *F(*$c_{i}^{1}$) = 1;4:  *F*($c_{i+k}^{1}$) = 1;5:
**end for**
6:**for ***t* = *i* + 1 to *i* + *k* − 1 **do**7:  max = −∞;8:  **for**
*s* = 1 to candidateList(*P_t_*).count **do**9:   *F*($c_{t}^{s}$) = *F*($c_{t-1}^{j}$) + *F*($c_{t-1}^{j}$ → $c_{t}^{s}$);10:   Alt = *F*($c_{t}^{s}$);11:   **if** (Alt > max) **then**12:   max = Alt;13:   *C* = max. $c_{t}^{s}$;14:   **end if**15:  **end for**16:  OSM-WayID-List.add(C.id);17:
**end for**


### 3.4. Trajectory Correction Algorithm

**Figure 9:**
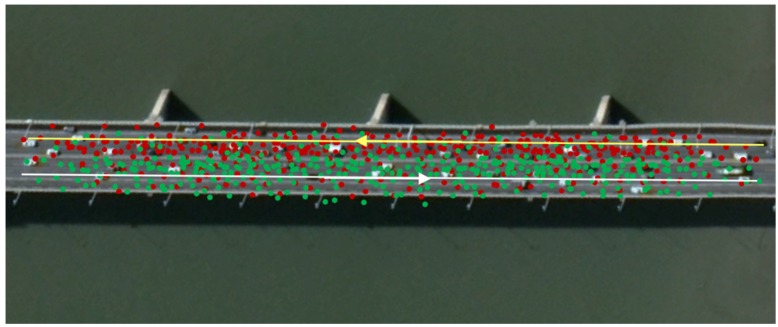
An example of the multipath effect. The yellow line is the OSM road from right to left and the white line is the OSM road from left to right. The red dots are matched to the yellow road and the green dots are matched to the white road. Some points appear on the wrong road because of the GPS error.

The accuracy of the trajectory improves after the optimization approach; however, it is not enough for the accurate generation of a lane-level map. As shown in Figure 9, the red points are matched to the yellow road which should locate at the road from right to left. However, some points appear on the opposite road because of the multipath effect, which will decrease the accuracy for generating the lane-level map. Thus, to address this issue, this paper proposes a physical attraction model based on the matched OSM map.

**Figure 10:**
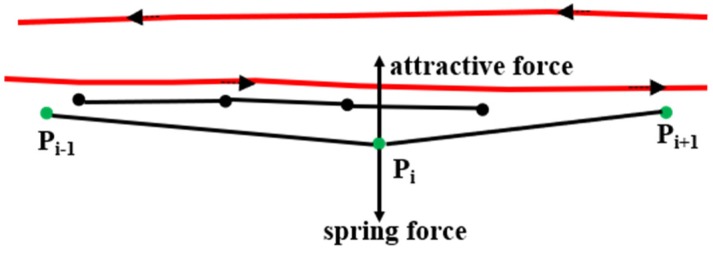
Two types of forces acting on the trajectories. The green dots are the GPS points on a trajectory and the black dots are the points on another trace.

According to [7,30], in the physical attraction model two types of forces act on the trajectories. One is an attractive force from the other traces in the same direction and on the same road. The other is a spring force to prevent the trace from moving away from its original position, as shown in Figure 10. All the traces in the same direction will be grouped together by these forces. The accuracy of this approach can reach the road level, but the original information of the trace is lost. Moreover, this approach makes mistakes at crossings, and it is time-consuming to calculate the distance between one trace and all the other traces. Thus, this paper introduces the matched OSM map algorithm to address these concerns.

**Figure 11:**
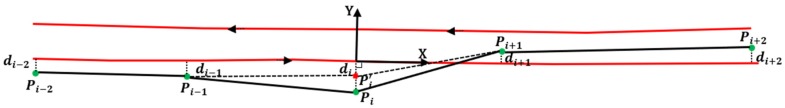
An example of the physical attraction model for point $P_{i}$. *P_i_* means the ID of GPS points, and d*_i_* represents the distance from GPS points to the matched OSM road. $P_{i}^{\prime }$ is the new position of *P_i_*. The green dots are the GPS points, and the red dot is the new point. The red lines are the OSM roads, the black lines represent the original trajectory, and the black dashed lines indicate the new trajectory.

In general, when a car drives on the road, it tends to keep driving in the same lane, unless an emergency occurs or it drives to an intersection. Therefore, the traces tend to keep the same distance with the matched OSM map. We calculated the attractive force by the relative distance between the trace and OSM road. Compared to calculating the distance between one trace and all the other traces, this method can greatly decrease the running time of the algorithm. At the intersection, our method is more reliable because the OSM map constrains the direction of the force. The equations are shown below:

(14) $$F_{1}\left(P_{i}\right)=-\frac{M}{\sigma^{3}\sqrt{2\pi }}e^{\left(-\frac{{\left(d_{i}-\bar{d}\right)}^{2}}{2\sigma^{2}}\right)}(d_{i}-\bar{d})$$

(15) $$F_{2}\left(P_{i}\right)=k\left(y-d_{i}\right)$$

(16) $$F_{1}\left(P_{i}\right)=F_{2}\left(P_{i}\right)$$

(17) $$\bar{d}=\frac{\sum_{i=1}^{n}d_{i}}{n},$$

where M and σ are the two experimental parameters that determine the potential energy of the attractive force, K is the spring constant, $d_{i}$ is the distance from $P_{i}$ to the matched OSM map points, $\bar{d}$ is the average distance of $d_{i}$, and $\left(y-d_{i}\right)$ is the difference of the distance between the new and the original position of point $P_{i}$ in order to keep $F_{1}\left(P_{i}\right)$ equal to $F_{2}\left(P_{i}\right)$. As shown in Figure 11, we set the direction of the OSM road as the x-axis and the left side of the road as the y-axis. $P_{i}^{\prime }$ is the new position of $P_{i}$. The details are shown in Algorithm 3.

**Algorithm 3** Physical Attraction Model
**Input:**
Trajectory *P*_1_ → *P*_2_ → *P_n_*; OSM-WayID-List;
**Output:**
New Trajectory $P_{1}^{\prime }$ → $P_{2}^{\prime }$ … → $P_{n}^{\prime }$;1:**for***t* = 1 to *n*
**do**2:  *T* = 0; *K* = ∞;3:  $\bar{d}$ = meandistance (*d*_1_, *d*_2_ … *d_n_*);4:  *K* = *d_i_* − $\bar{d}$;5:  **While**
*T* ≤ 20 && *K* > 0.5 **do**6:   *F*_1_(*P_i_*) = *F*_2_(*P_i_*);7:   ${\bar{d}}^{\prime }$ = meandistance ();8:   *K* = $d_{i}^{\prime }$ − ${\bar{d}}^{\prime }$;9:   *T* = *T* + 1;10:  **end while**11:
**end for**


## 4. Experimental Tests of the Proposed Approach

### 4.1. Experimental Data

**Figure 12:**
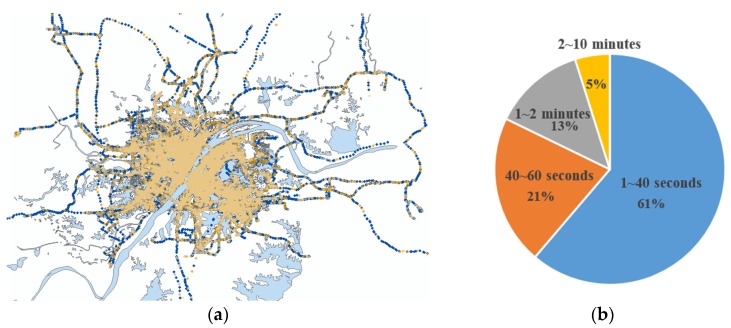
The experimental data. (**a**) GPS points collected from taxis; the yellow dots represent the GPS points whose sampling frequency ranged from 2–10 min; the gray dots are the points who sampling frequency ranged from 1–2 min; the orange dots are the points whose sampling frequency ranged from 40–60 s; and the blue dots are the points whose sampling frequency ranged from 1–40 s. (**b**) Distribution of the sampling frequencies.

To test the algorithms proposed in this paper, we collected about 40 million GPS points from thousands of taxis within one week in Wuhan, China. The sampling frequency ranged from 1 s to 10 min, as shown in Figure 12. Over 60% of the points are included in the sampling frequency range of 1–40 s.

### 4.2. Trajectory Preprocessing

**Figure 13:**
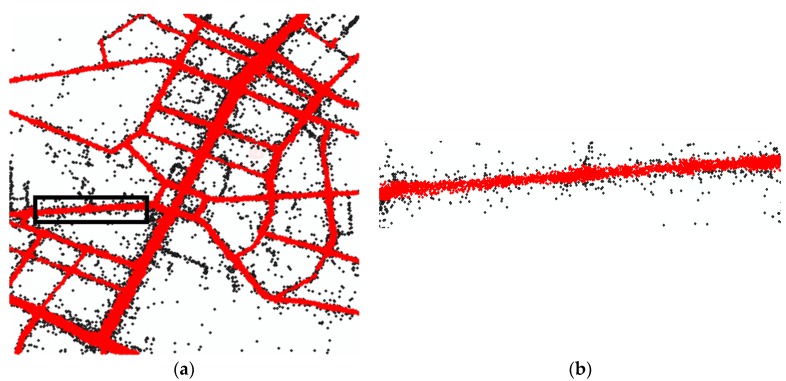
The results of preprocessing. The red dots are the preserved GPS points and the black dots are noise points (**a**) An example on a large scale. (**b**) The detailed results of the black rectangle in (**a**).

The trajectory preprocessing algorithm is an adaptive density optimization method. The result is shown in Figure 13. The red dots and black dots represent the selected GPS points and outliers, respectively; the noise points that are removed by this method.

### 4.3. Map Matching

**Figure 14:**
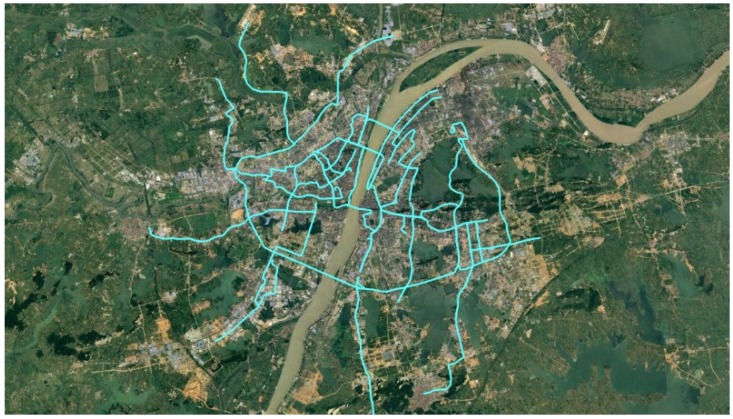
The labeled trajectories. The teal lines represent the labeled traces.

The matching data was labeled by real people. Compared to the synthetic trajectory data used in reference [17], it is more reliable. The labeled data contains 34 traces covering about 494 km, as represented in Figure 14.

#### 4.3.1. Evaluation Approach

To evaluate the matching quality, we calculated the accuracy and recall, as shown in the following equations:

(18) $$accuracy=\frac{Number\;of\;correctly\;matched\;points}{Total\;number\;of\;matched\;points}\times 100\%$$

(19) $$recall=\frac{Number\;of\;correctly\;matched\;points}{Number\;of\;relavant\;points\;in\;labeled\;points}\times 100\%$$

#### 4.3.2. Parameter Selection

**Figure 15:**
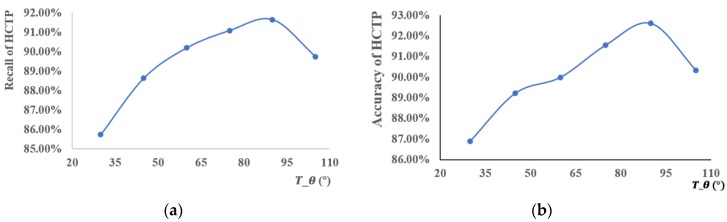
The (**a**) recall and (**b**) accuracy of map matching for different $T_{\theta }$. The blue lines represent the value of recall and accuracy of HCTP for different $T_{\theta }$.

In Section 3.3.1, we used the threshold $T_{\theta }$ to screen out the candidate segments. The accuracy and recall are different, depending on the choice of $T_{\theta }$, as shown in Figure 15. The recall of the HCTPs is about 86–92%, and the points matched as HCTP do not need to be matched further. This reduces the running time of the algorithm. The accuracy of HCTP is about 90%. This means that Assumption 1, as proposed above, is dependable. According to these results, when we set $T_{\theta }$ = 90° the accuracy and recall reach their maximums.

In the ST-matching step, the parameters included the following: $\mu ,\;\sigma ,\;w_{1},\;w_{2},\;\tau ,\;R.\sigma ,\;R.u$. μ and σ are the mean value and variance of the normal distribution, respectively. In this paper, we set μ=0 and σ=10. $w_{1}$ and $w_{2}$ are the weight of the observation and the transmission probabilities, respectively. As explained above, the transmission probability is more reliable than the observation probability; therefore, we set $w_{1}=0.3\;\mathrm{and}\;w_{2}=0.7$. The value of τ was 10 km/h. R.u and R.σ are the mean value and variance of the road speed constraints, respectively. The values for the different roads are shown in Table 2.

#### 4.3.3. Matching Result

**Figure 16:**
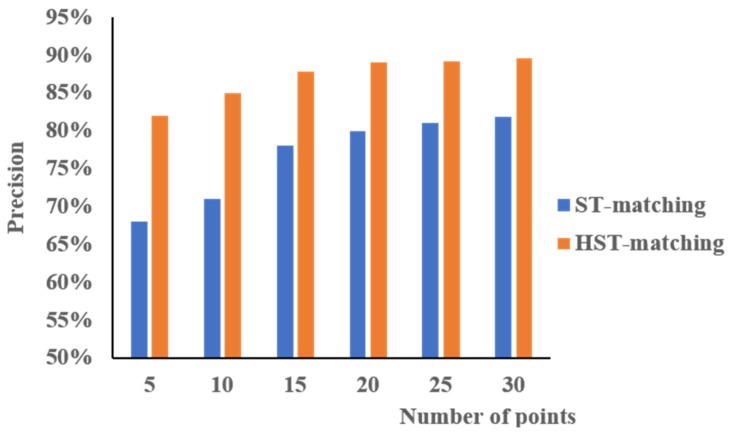
The accuracy of map matching using spatial–temporal (ST)-matching (blue) vs. hierarchical ST (HST)-matching (orange).

We compared the HST-matching results with those of the ST-matching algorithm. To evaluate the quality and efficiency of the two algorithms, we compared the accuracy and running time. Figure 16 represents the accuracy comparison results. When the number of points on a trajectory was in the range 5–15, the HST-matching algorithm significantly outperformed the ST-matching algorithm; the accuracy of the HST-matching algorithm showed about a 15% improvement. With an increasing number of points in a trajectory, the performance of these two algorithms became more similar, but the HST-matching algorithm still showed about an 8% improvement over the ST-matching algorithm.

#### 4.3.4. Running Time

**Figure 17:**
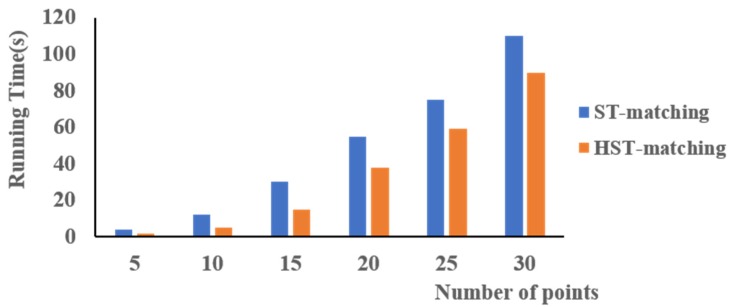
Running time of the map matching using ST-matching (blue) vs. HST-matching (orange).

As shown in Figure 17, it is clear that the HST-matching algorithm is faster than the ST-matching algorithm, especially when the number of points is in the range 5–15. This is because we calculate the HCTPs first. This method greatly reduces the number of unnecessary calculations, especially when there are fewer points on the trajectory. As the number of points increases, the time cost of the two methods increases quickly and tends to become more similar. This is because the algorithms need more time to calculate the shortest path with an increased number of points.

### 4.4. Data Correction

#### 4.4.1. Parameter Selection

In this part, there are three main parameters that need to be set: M,σ,and k. According to reference [30], we set M=1, σ=10, and *k* = 0.005.

#### 4.4.2. Correction Result

**Figure 18:**
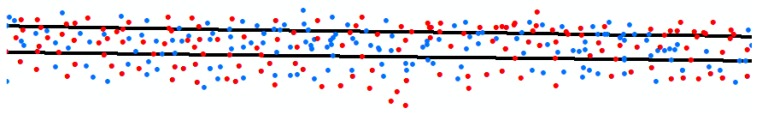
Original data from a road. The black lines represent the OSM road, the red dots represent the points matched to the upper black line, and the green dots are the points matched to the lower line. The points from different directions are mixed together.

**Figure 19:**
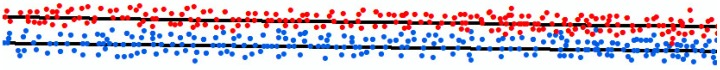
The results of the correction algorithm represented in this paper. The points from different directions separate well.

Figure 18 shows the original data. The data is messy, and there are a many points that appear on the wrong side of the road, against the traffic regulations. After the data correction algorithm proposed in this paper is used the position accuracy of the data improved. The trajectory no longer appears in the opposite lane; the points corrected to the right position, as shown in Figure 19.

**Figure 20:**
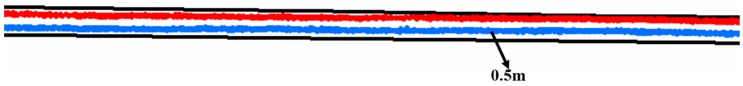
The results of the algorithm represented in reference [30]. The data with the same direction clustered together, and the maximum gap was 0.5 m.

**Figure 21:**
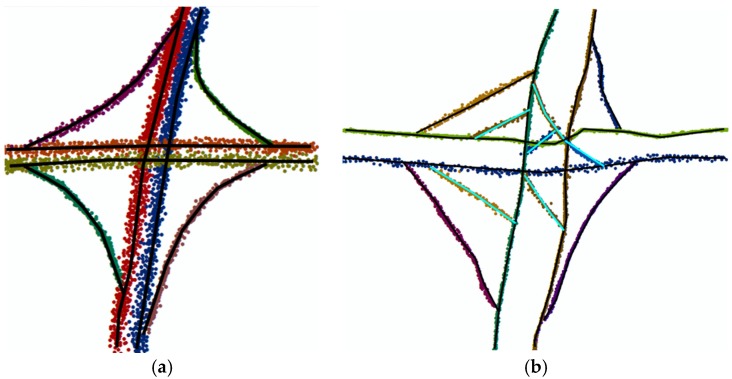
An example of an intersection. (**a**) The result of the algorithm represented in this paper. Various colors represent different directions of GPS points. (**b**) The result of the algorithm represented in reference [30], in which there are some incorrect edges as represented by the teal lines.

The algorithm proposed in reference [30] groups the traces with the same direction together; the gap between them is less than 0.5 m, as represented in Figure 20. By this approach, the accuracy of the data can only reach the road level, beyond which a lane-level map cannot be generated. Additionally, original information of the floating car traces is lost. Moreover, there are still some mistakes at the intersections when using this approach, as shown in Figure 21b; there are some incorrect edges that need to be removed. In contrast, in our method points are previously matched to the OSM, so they can be correctly clustered especially at the intersection, as shown in Figure 21a.

The time complexity of the algorithm in reference [30] is (M^2^), where M is the number of nodes in the GPS dataset. For each node, a dataset was a square (100 × 100 m) centered at the node. It took at least 15 s to calculate the data for each node. However, the time complexity of the algorithm proposed in this paper is (M). Our algorithm only needs 150 ms to calculate the data for each point—a marked improvement on previous algorithms.

## 5. Conclusions

In this paper, we proposed a data correction algorithm for low-frequency floating car data. After preprocessing the data, we employed an HST-matching algorithm to match the GPS trajectories with the OSM map. The accuracy and running time of this algorithm were compared with those of the ST-matching algorithm. The accuracy of the HST-matching algorithm was higher; the accuracy of the HST-matching algorithm was always 8–15% higher than that of the ST-matching algorithm. Moreover, we needed less time to calculate the results because we adopted a hierarchical algorithm to calculate the HCTP first. Next, the data was corrected by a physical attraction model based on the matched OSM map. A verification experiment was conducted based on the data of actual taxi trajectories. The results showed that the accuracy of the data after the correction was improved, especially at the crossroads. Moreover, we improved the time efficiency by 150 times.

This paper proved that OSM can be used to improve the accuracy of low-floating car data. This study was also useful for increasing the precision of the production of lane-level maps, which were generated by the corrected data.

However, although we greatly improved the time efficiency, it still took a long time to calculate all the data because of the huge quantity of floating car data. In the future, we will continue to improve the calculation efficiency of this algorithm and to research the production of lane-level maps.

## Figures and Tables

**Table 1:** The road speed constraint ranges in China.

Value	Motorway	Trunk	Primary	Secondary	Tertiary	Service	Residential
Min-speed (km/h)	90	60	40	30	20	0	0
Max-speed (km/h)	120	100	60	50	40	20	15

**Table 2:** The mean (R.u) and variance (R.σ ) of the road speed constraints for the different roads.

Threshold	Motorway	Trunk	Primary	Secondary	Tertiary	Service	Residential
R.u	105	80	50	40	30	10	10
R.σ	5	7	3	3	3	3	1.5
